# T80. CALCIUM AND POTASSIUM VOLTAGE-GATED CHANNELS GENES ASSOCIATION ANALYSIS: EVIDENCE ON THEIR ROLE IN COGNITIVE PERFORMANCE OF SCHIZOPHRENIA PATIENTS AND HEALTHY SUBJECTS

**DOI:** 10.1093/schbul/sby016.356

**Published:** 2018-04-01

**Authors:** Maria Guardiola, Carmen Almodóvar, Alba Lubeiro Juárez, Silvia Alonso-Lana, Jordi Ortiz-Gil, Amalia Guerrero-Pedraza, Salvador Sarró, Edith Pomarol-Clotet, Mar Fatjó-Vilas

**Affiliations:** 1 University of Barcelona; 2 School of Medicine, University of Valladolid; 4 FIDMAG Research Foundation, CIBERSAM; 5 FIDMAG Research Foundation, Hospital General de Granollers; 6 Benito Menni - CASM, Sant Boi de Llobregat; 7 FIDMAG Research Foundation, CIBERSAM, University of Barcelona

## Abstract

**Background:**

Cognitive deficits are considered core features of schizophrenia (SZ) (Green & Harvey 2014). Subtle variations in the perfectly coupled mechanism that maintains the potential of membrane in neurons may have repercussion on neuronal processing. Therefore, genetic variability related to the functioning of excitable cells and linked to pathways essential for neuronal survival and plasticity may underlie the observed differences in cognitive abilities (Carr et al 2016). CACNA1C and KCNH2 genes encode for calcium and potassium voltage-gated channels, ultimately related to neuronal functioning (Dolmetsch et al 2001, Schwarz et al 2004). These two genes have been previously related with SZ (Atalar et al 2010, Ripke et al 2014). The aim of this study was to evaluate whether the genetic variability of CACNA1C and KCNH2 is associated with: i) the risk for schizophrenia, ii) the cognitive performance of SZ patients and healthy subjects.

**Methods:**

Our sample consisted of 348 SZ patients and 387 unrelated healthy controls (HC). DNA was extracted from blood/saliva samples using standard procedures and two Single Nucleotide Polymorphisms (SNPs) were genotyped: rs1006737 (G/A) in CACNA1C gene, rs3800779 (G/A) in KCNH2. A subsample (296 SZ/157 HC) underwent neurocognitive assessment, which included: i) premorbid IQ (Word Accentuation Test - Test de Acentuación de Palabras (TAP)); ii) memory (Wechsler Memory Scale (WMS-III)) and, iii) executive function (Behavioural Assessment of the Dysexecutive Syndrome (BADS)). The association between the SNPs and neurocognitive performance was explored (adjusted by sex and age) separately in patients and in controls groups.

**Results:**

In our sample, we did not detect an association of CACNA1C and KCNH2 with the risk for SZ. Patients performed significantly worse than controls in all cognitive measures (p<0.005). SZ patients homozygous for the risk allele (A) of the CACNA1C polymorphism showed lower premorbid IQ (TAP scores) than patients carriers of the C allele (rs1006737: B=-1.39 p=0.027). Within HC, the minor allele (A) of KCNH2 was associated with WMS global score (rs3800779: B=3.01 p=0.010): subjects carrying the AA genotype presented better memory performance.

**Discussion:**

Our findings add evidence on the role of CACNA1C and KCNH2 on modulating cognitive performance in SZ patients and HC (Huffaker et al 2009, Krug et al 2010, Zhang et al 2012, Hashimoto et al 2013). Our results in patients are in line with previous studies that suggest an association of CACNA1C risk allele on different cognitive domains. As regards to KCNH2, our results are opposite in terms of the direction of the effect observed in previous studies, probably as a consequence of the sample size and heterogeneity in methods used to assess memory. The different direction of the genetic effects among patients and controls reflects the complex relationship between genetic factors and cognitive performance variability. It is suggested that genes that enhance cognitive abilities under normal circumstances turn to be pernicious under the modulation effect of a disease (Crespi et al 2007). Further research is needed and we expect to extend the present results with neuroimaging genetics approaches.

**Acknowledegments:**

Spanish Ministry of Economy and Competitivity, Instituto de Salud Carlos III (PI15/01420 and PI15/00299), Ayuda cofinanciada por el Fondo Europeo de Desarrollo Regional (FEDER) “Una manera de hacer Europa”.

